# Sunitinib added to FOLFIRI versus FOLFIRI in patients with chemorefractory advanced adenocarcinoma of the stomach or lower esophagus: a randomized, placebo-controlled phase II AIO trial with serum biomarker program

**DOI:** 10.1186/s12885-016-2736-9

**Published:** 2016-08-31

**Authors:** Markus Moehler, Irina Gepfner-Tuma, Annett Maderer, Peter C. Thuss-Patience, Joern Ruessel, Susanna Hegewisch-Becker, Hansjochen Wilke, Salah-Eddin Al-Batran, Mohammad-Reza Rafiyan, Florian Weißinger, Hans-Joachim Schmoll, Frank Kullmann, Ludwig Fischer von Weikersthal, Jens T. Siveke, Jens Weusmann, Stephan Kanzler, Carl Christoph Schimanski, Melanie Otte, Lukas Schollenberger, Jochem Koenig, Peter R. Galle

**Affiliations:** 1 University Medical Center, Johannes Gutenberg-University Mainz, I. Medizinische Klinik und Poliklinik, Langenbeckstraße 1, 55131 Mainz, Germany; 2 Charité Campus Virchow-Klinikum, Berlin, Germany; 3 University Hospital Halle (Saale), Halle (Saale), Germany; 4 Hämatologisch-Onkologische Praxis Eppendorf, Hamburg, Germany; 5 Kliniken Essen-Mitte - Evang. Huyssens-Stiftung, Essen, Germany; 6 Krankenhaus Nordwest, Frankfurt, Germany; 7 Evangelisches Krankenhaus Bielefeld, Bielefeld, Germany; 8 Kliniken Nordoberpfalz - Klinikum Weiden, Weiden, Germany; 9 Gesundheitszentrum St. Marien GmbH, Amberg, Germany; 10 Klinikum rechts der Isar, Technical University Munich, Munich, Germany; 11 Leopoldina Krankenhaus, Schweinfurt, Germany; 12 Marienkrankenhaus Darmstadt, Darmstadt, Germany; 13 Praxisgemeinschaft für Onkologie und Urologie, Wilhelmshaven, Germany; 14 Interdisciplinary Center for Clinical Trials (IZKS), Mainz, Germany; 15 Institute of Medical Biostatistics, Epidemiology and Informatics (IMBEI) of the University Medical Center Mainz, Mainz, Germany

**Keywords:** Chemorefractory advanced gastric cancer, Tyrosine kinase inhibitor, Sunitinib, FOLFIRI, VEGF

## Abstract

**Background:**

As a multi-targeted anti-angiogenic receptor tyrosine kinase (RTK) inhibitor sunitinib (SUN) has been established for renal cancer and gastrointestinal stromal tumors. In advanced refractory esophagogastric cancer patients, monotherapy with SUN was associated with good tolerability but limited tumor response.

**Methods:**

This double-blind, placebo-controlled, multicenter, phase II clinical trial was conducted to evaluate the efficacy, safety and tolerability of SUN as an adjunct to second and third-line FOLFIRI (NCT01020630). Patients were randomized to receive 6-week cycles including FOLFIRI plus sodium folinate (Na-FOLFIRI) once every two weeks and SUN or placebo (PL) continuously for four weeks followed by a 2-week rest period. The primary study endpoint was progression-free survival (PFS). Preplanned serum analyses of VEGF-A, VEGF-D, VEGFR2 and SDF-1α were performed retrospectively.

**Results:**

Overall, 91 patients were randomized, 45 in each group (one patient withdrew). The main grade ≥3 AEs were neutropenia and leucopenia, observed in 56 %/20 % and 27 %/16 % for FOLFIRI + SUN/FOLFIRI + PL, respectively. Median PFS was similar, 3.5 vs. 3.3 months (hazard ratio (HR) 1.11, 95 % CI 0.70–1.74, *P* = 0.66) for FOLFIRI + SUN vs. FOLFIRI + PL, respectively. For FOLFIRI + SUN, a trend towards longer median overall survival (OS) compared with placebo was observed (10.4 vs. 8.9 months, HR 0.82, 95 % CI 0.50–1.34, one-sided *P* = 0.21). In subgroup serum analyses, significant changes in VEGF-A (*P* = 0.017), VEGFR2 (*P* = 0.012) and VEGF-D (*P* < 0.001) serum levels were observed.

**Conclusions:**

Although sunitinib combined with FOLFIRI did not improve PFS and response in chemotherapy-resistant gastric cancer, a trend towards better OS was observed. Further biomarker-driven studies with other anti-angiogenic RTK inhibitors are warranted.

**Trial registration:**

This study was registered prospectively in the NCT Clinical Trials Registry (ClinicalTrials.gov) under NCT01020630 on November 23, 2009 after approval by the leading ethics committee of the Medical Association of Rhineland-Palatinate, Mainz, in coordination with the participating ethics committees (see Additional file 2) on September 16, 2009.

**Electronic supplementary material:**

The online version of this article (doi:10.1186/s12885-016-2736-9) contains supplementary material, which is available to authorized users.

## Background

Overall survival (OS) for patients with locally advanced and metastatic gastric cancer (AGC) remains poor with a median OS of 8–11 months [1–3]. Many clinical trials have investigated novel first-line chemotherapeutic combinations without demonstrating a clear survival benefit compared with standard regimens [1, 4, 5]. Several prospective randomized trials indicate a significant increase in OS for second-line chemotherapy compared with best supportive care (BSC) [6, 7]. Although none of the investigated combination regimens demonstrated significant advantages over the others, the combination of irinotecan, 5-fluorouracil (5-FU) and folinic acid (FOLFIRI) is accepted as a safe and efficient chemotherapeutic treatment for patients with refractory AGC [2].

Tumor angiogenesis, growth and metastasis can be inhibited by blocking receptor tyrosine kinases (RTKs) which are known to be overexpressed in human gastric cancer (GC), including vascular endothelial growth factor receptors (VEGFRs) or platelet-derived growth factor receptors (PDGFRs) [3, 8]. Furthermore, disease progression or poor survival is associated with VEGF-A, epithelial growth factor receptor (EGFR) and PDGF-A expression in the tumor [9, 10]. Serum VEGF levels are significantly higher in GC patients with remaining tumor and decrease with radical resection. High preoperative serum VEGF-A correlate with poor survival, tumor invasion and distant metastases [11]. In contrast, in GC patients treated with the VEGF-A antibody bevacizumab high plasma VEGF-A predicted improved OS [12, 13]. Interestingly, the prognostic significance of VEGF-A concentrations could be demonstrated for non-Asian population only [12, 14]. Additionally, a correlation of VEGF expression in GC tumor tissue with histopathological response was shown [11, 13, 15, 16].

Agents such as gefitinib, erlotinib and cetuximab specifically target RTKs through a dominant receptor pathway and have been investigated in phase II-III studies in patients with AGC [17]. However, in many tumors, several RTKs are co-expressed [8]. In addition, the initiation of alternative angiogenesis signaling pathways under therapy with anti-angiogenic agents represents a potential cause for therapy resistance of tumor cells [18–20]. Stromal cell-derived factor-1 (SDF-1α), also known as CXCL12, may induce proliferation, dissemination and immune evasion of several tumor tissues with several existing variants [18, 19, 21–23]. However, studies investigating its expression in GC specimens have revealed inconsistencies regarding its occurrence in cancer tissue and its correlation with clinical characteristics [24, 25].

Sunitinib malate (SUN) is an oral, multi-targeted RTK inhibitor of VEGFR-1, −2 and −3, PDGFR-α and -β and several other RTKs [26, 27] and may have additional benefits compared to single receptor targeted inhibition. In two recent phase II studies in patients with chemorefractory AGC, SUN showed promising activity and manageable toxicity [28, 29]. Therefore, this phase II clinical trial was conducted to evaluate the efficacy, safety and tolerability of SUN versus placebo (PL) as add-on therapy to the second-line FOLFIRI treatment regimen.

## Methods

### Study design and treatment

This randomized, double-blind, placebo-controlled, multicenter, phase II study was conducted at 15 sites in Germany after approval of the leading and the local ethics committees. It was performed according to the International Conference on Harmonisation – Good Clinical Practice and the Declaration of Helsinki and was registered in the public NCT Clinical Trials Registry (ClinicalTrials.gov) under NCT01020630.

After signing informed consent, patients (SUN-CASE inclusion/exclusion criteria: Additional file 1: Table S1) were randomized to receive either SUN or PL in addition to the two-weekly FOLFIRI plus sodium folinate (Na-FOLFIRI) chemotherapy backbone. Patients were treated until occurrence of any of the following: progressive disease, intolerable adverse events (AEs), any AE that resulted in treatment interruption of >14 days within the active treatment cycle or >4 weeks between consecutive active treatment cycles, or withdrawal of consent.

SUN (starting dose: 25 mg) or PL was administered orally once daily for four consecutive weeks followed by a 2-week rest period. The dose of SUN could be increased to 37.5 mg at the discretion of the treating physician and upon approval by the Data and Safety Monitoring Board (DSMB) in patients tolerating 25 mg in the first cycle without dose-limiting toxicities. The dose could be reduced to 12.5 mg in patients experiencing SUN-related toxicity.

FOLFIRI was administered as the following regimen: Irinotecan (180 mg/m^2^) was given intravenously on day 1, immediately followed by 5-FU bolus (400 mg/m^2^) and 46-h infusion of sodium folinate (400 mg/m^2^) and 5-FU (2000 mg/m^2^) every two weeks, i.e. three courses of FOLFIRI in every 6-week SUN/PL cycle [30]. Treatment continued until disease progression or occurrence of unacceptable toxicity/AEs.

### Biomarker analysis

An important inclusion criterion was the existance of at least one blood sample before first medication intake (baseline) and a further available sample during the study. The peripheral blood samples were collected at all 15 participating study centers. After collection, specimens were centrifuged to separate the serum which was stored at −80 °C. Serum samples were tested in duplicate for concentrations of VEGF-A, VEGF-D, soluble VEGFR2 and SDF-1α on days 1 + 14 of the first cycle. Furthermore, premedication serum samples from day 1 of cycle 2 and cycle 3 were evaluated. The following enzyme-linked immunosorbent assays (Duo-ELISA - R&D, Minneapolis) were performed for quantification of serum levels using standard curves for concentration calculation: Duoset® ELISA Human VEGF (DY293B), Duoset® ELISA Human VEGF-D/FIGF (DY622), Duoset® ELISA Human VEGFR2/KDR (DY357) and Duoset® ELISA Human CXCL12/SDF1 (DY350) according to manufacturer’s protocols.

### Safety and efficacy assessment

AEs were graded according to the National Cancer Institute (NCI) Common Terminology Criteria for AEs (CTCAE), version 4.0.

Tumor response was measured by computed tomography scan after cycle 1 and 2, then after every 2 cycles, assessed and graded by RECIST 1.1. Screening assessments were carried out within 28 days prior to the start of treatment. After the end of treatment (EOT), an EOT visit was performed within 30 days. Patients were followed-up every 3 months for 1 year thereafter.

### Trial objectives and statistical analysis

The primary endpoint was progression-free survival (PFS) according to RECIST 1.1. Secondary endpoints were objective response rate, tumor control rate (complete response [CR] + partial response [PR] + stable disease [SD]), duration of disease stabilization, 1-year OS, and the safety and tolerability of the placebo-controlled combination therapy compared to the standard second-line therapy.

In total, 90 patients were to be enrolled to assign 43 patients to each treatment arm, taking into account a drop-out rate of 5 %. A median PFS of 3 months was assumed for the control group. A total of 86 events had to be observed to show a 50 % improvement (4.5 months median PFS) under SUN versus PL to ensure a power of 80 %, at a one sided significance level α of 15 %.

All statistical analyses were performed using SPSS® Statistics. Measured biomarker values were partially log-transformed for statistical purposes. Kaplan-Meier analysis with log-rank test was performed to estimate PFS and OS. Cox proportional hazards model was used for survival and covariates analyses. Proportions of adverse events were compared by chi-square test or Fisher’s exact test if the event occurred in less than 10 patients. Non-parametric Wilcoxon-tests were used to compare serum levels between different samples. To assess correlations between serum levels and clinical parameters, Spearman coefficients, non-parametric Mann–Whitney and Jonckheere-Terpstra tests were performed. Tests with *P* < 0.05 were considered statistically significant.

The primary analysis population was the intention-to-treat (ITT) set comprising all patients with at least one available post-baseline assessment of the primary analysis variable. The safety analysis included all patients who had received at least one dose of trial medication. The analysis of secondary endpoints and all further data were interpreted descriptively.

## Results

### Patient characteristics

**Fig. 1:**
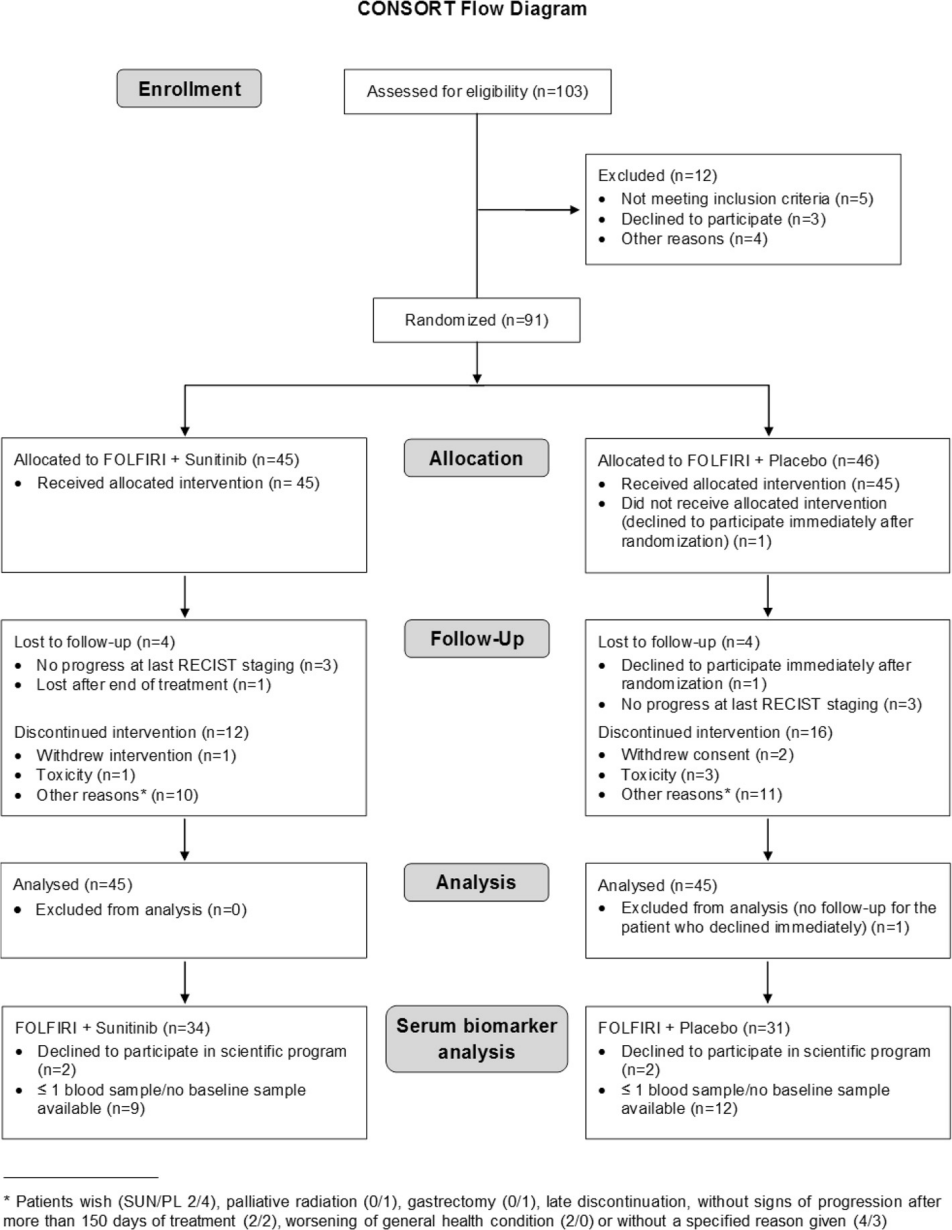
Consort flow diagram

Overall, 91 patients were enrolled (Fig. 1). One patient withdrew consent immediately after randomization and was not included in the ITT analysis (SUN/PL 45/45).

Demographic and baseline characteristics for the ITT patient population compared with the evaluable population for serum analysis are provided in Table 1.

**Table 1:** Demographic, baseline and response characteristics in the ITT population and serum analysis subpopulation

Characteristic	ITT population				Serum analysis population			
	Sunitinib		Placebo		Sunitinib		Placebo	
	*N*	*n* (%)	*N*	*n* (%)	*N*	*n* (%)	*N*	*n* (%)
Number of patients	45	100	45	100	34	100	31	100
Age (years)								
Mean (SD)	59 (11)		57 (11)		57 (12)		58 (12)	
Median (Range)	62		57		60		60	
	(37–76)		(28–84)		(37–76)		(28–84)	
Gender								
Male	33	73	30	67	25	74	18	58
Female	12	27	15	33	9	26	13	42
Karnofsky performance status								
90–100 %	27	60	26	58	22	65	19	61
70–80 %	16	36	18	40	10	29	11	25
Not known	2	4	1	2	2	6	1	3
Histology: Adenocarcinoma of								
Stomach	22	49	23	51	17	50	17	55
Cardia	23	51	20	44	17	50	14	45
Not known	0	0	2	4	0	0	0	0
Treatment lines before study entry								
1	34	76	34	76	26	76	24	77
2	9	20	11	24	6	18	7	23
3	1	2	–	–	2	6	–	–
Not known	1	2	0	0	0	0	0	0
Screening pT-stadium								
0	–	–	1	2	–	–	1	3
1	1	2	1	2	1	3	1	3
2	5	11	7	16	4	12	6	19
3	21	47	18	40	17	50	9	29
4	7	16	9	20	6	18	7	23
X	11	24	9	20	6	18	7	23
Screening pN-stadium								
0	2	4	7	16	1	3	6	19
1	16	36	11	24	16	47	7	23
+	–	–	1	2	–	–	–	–
2	9	20	8	18	7	21	7	23
3	8	18	6	13	7	21	5	16
X	10	22	12	27	3	9	6	19
Screening pM-stadium								
0	9	20	4	9	8	24	3	10
1	36	80	41	91	26	77	28	90
Best response								
Complete response (CR)	–	–	5	11	–	–	4	13
Partial response (PR)	9	20	8	18	8	24	6	19
Stable disease (SD)	18	40	12	27	17	50	9	29
Progressive disease (PD)	14	31	16	36	8	24	12	39
Not evaluable	4	9	4	9	1	3	–	–
Objective response (CR + PR)	9	20	13	29	8	24	10	32
Tumor control rate (CR + PR + SD)	27	60	25	56	25	74	19	61

*SD* standard deviation

Follow-up for progressive disease (PD) was carried out at 3, 6, 9 and 12 months (±2 weeks) after the EOT visit until progression. Progression was observed in 32 and 31 patients of the SUN and PL groups, respectively. In 6/9 of SUN/PL patients, respectively, progression was observed >12 months after the EOT visit. At the end of the study, 7/5 of SUN/PL patients were reported to have no signs of PD.

### Treatment

Patients in both groups started 2.7 cycles of treatment. In total, 29 and 24 patients from the SUN and PL groups respectively, terminated treatment due to disease progression. Further reasons for ending treatment (SUN/PL) were treatment interruption (3/2 patients), toxicity (1/3 patients) and withdrawal of informed consent (1/2 patients).

### Efficacy

**Fig. 2:**
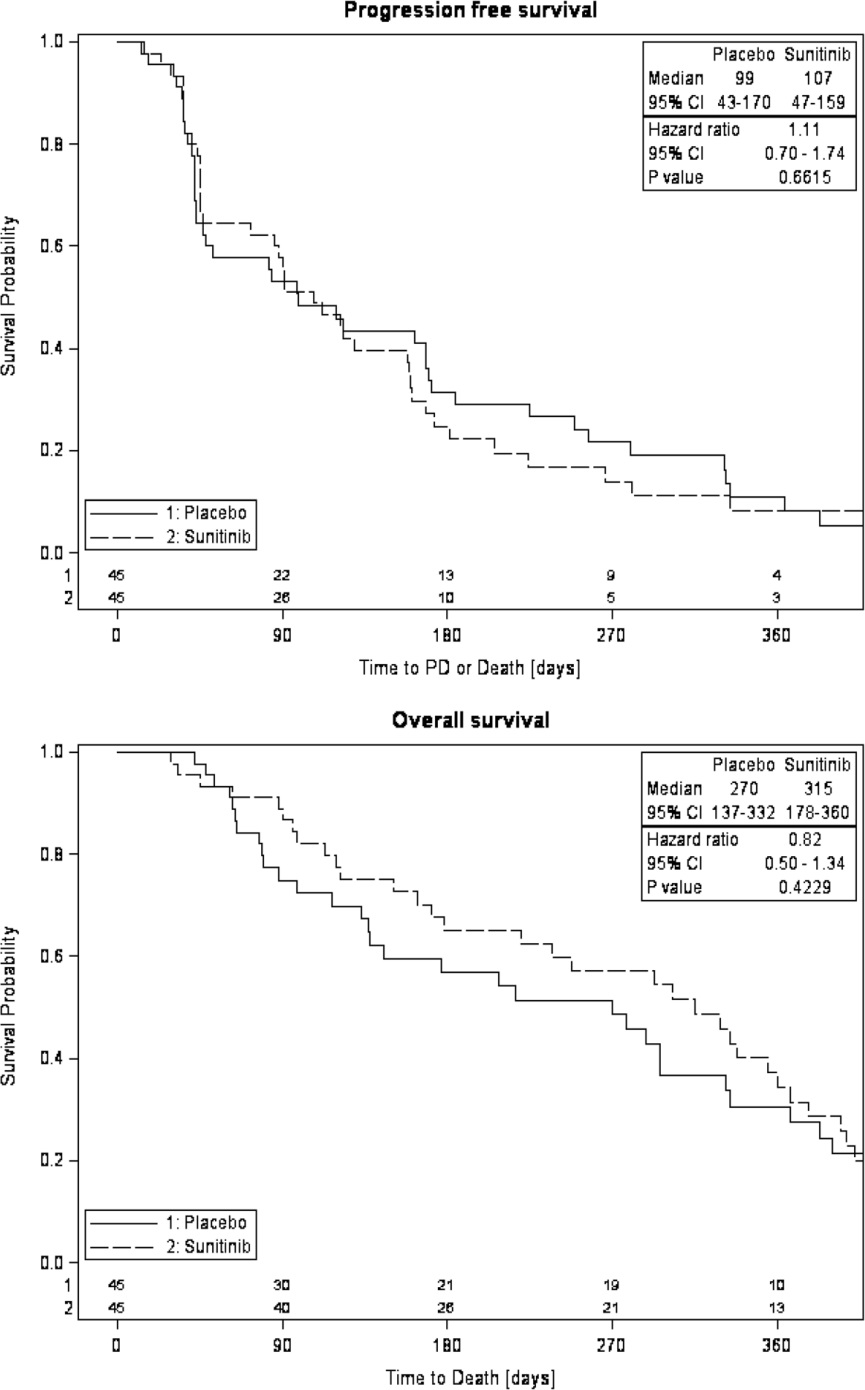
Kaplan-Meier curves for progression-free survival and overall survival in the primary analysis population (ITT). Hazard ratios estimated by Cox proportional hazards model. PFS, progression-free survival; HR, hazard ratio; ITT, intention-to-treat

Efficacy analysis was carried out on the ITT population. Figure 2 illustrates the survival distribution per treatment group for PFS and OS by Kaplan-Meier curves. The median PFS was similar in both groups, 107 and 99 days (3.5 vs. 3.3 months) for SUN and PL patients, respectively (HR 1.11, 95 % CI 0.70–1.74, *P* = 0.66). The OS showed a trend in favor of SUN compared with PL, 315 vs. 270 days (10.4 vs. 8.9 months). However, the difference was not statistically significant (HR 0.82, 95 % CI 0.50–1.34, *P* = 0.42). The probability of 1-year survival was 34 % and 36 %, and the probability of living 180 days was 0.65 and 0.57 for the SUN and PL groups, respectively.

Evaluation of response and tumor control was performed in the ITT population (Table 1). Best responses according to RECIST 1.1 were defined in 79 patients (Table 1). Objective response and tumor control were achieved in 20 %/29 % and 58 %/56 % for FOLFIRI + SUN/FOLFIRI + PL, respectively. Disease progression was the cause of death in 32 % of patients treated with SUN and in 31 % of PL patients.

### Safety and tolerability

Overall, 43 SUN and 42 PL patients experienced at least one AE. In total, 33 SUN and 31 PL patients had at least one AE CTC-Grade >3. CTC-Grade 4 and Grade 5 AEs were reported in 15/7 and 3/5 of SUN/PL patients, respectively. The frequencies of grade ≥3 AEs are shown in Table 2.

**Table 2:** Frequency of adverse events grade ≥ 3, reported in ≥ 2 (4 %) of patients of either group regardless of causality

Adverse events	Sunitinib plus FOLFIRI^a^		Placebo plus FOLFIRI^a^	
	*N* = 45	100 %	*N* = 45	100 %
Neutropenia	25	56	9	20
Leucopenia	12	27	7	16
Diarrhea	1	2	6	13
Nausea	3	7	3	7
Vomiting	3	7	3	7
Fatigue	-	-	4	9
Pain	-	-	4	9
Pulmonary embolism	2	4	2	4
General physical health deterioration	2	4	2	4
Mucosal inflammation	2	4	2	4
Gamma-glutamyltransferase increased	3	7	1	2
Pneumonia	-	-	3	7
Subileus	-	-	3	7
Blood acid phosphatase increased	2	4	-	-
Blood bilirubin increased	2	4	-	-

*FOLFIRI* 5-fluorouracil, leucovorin and irinotecan

^a^Schedule: 4/2, 4 weeks on treatment, followed by 2 weeks off; dosage: starting dose 25 mg/day

Neutropenia: *p* < 0.001, leucopenia *p* = 0.20, all other items statistically not significant (Fisher’s exact test)

AEs of Grade ≥3 at least possibly related to study medication comprised neutropenia (24/8), leucopenia (11/5), diarrhea (0/4), mucosal inflammation (2/2), fatigue (0/3), pulmonary embolism (2/1), nausea (1/2), and vomiting (2/1) for SUN/PL patients, respectively. With the notable exception of neutropenia (*P* < 0.001), all proportions of patients exhibiting an AE were statistically not significant. No unexpected toxicities/AEs occurred with SUN.

### Biomarker analysis

No significant correlations of any biomarker levels to age, gender, lymph node or distant metastases were found. Similar PFS and OS results in the observed subgroup and the ITT population, suggest an absence of bias. Tables 3 and 4 summarize the main findings.

**Table 3:** Effects of study medication on biomarker serum levels

Serum biomarker and study group		Level changes^a^					
		Δδ day 14 of cycle 1 - baseline	*P* value	Δδ day 1 of cycle 2 - baseline	*P* value	Δδ day 1 of cycle 3 - baseline	*P* value
VEGF-A	Sunitinib	-0.6816 (1.43)	**0.017**	-0.1393 (1.83)	0.831	0.1111 (1.26)	0.975
	Placebo	-0.3134 (1.24)	0.472	-0.7184 (1.32)	**0.033**	-1.1177 (1.92)	0.062
sVEGFR2	Sunitinib	-0.0648 (0.17)	**0.012**	-0.0854 (0.12)	**0.006**	-0.0727 (0.14)	0.078
	Placebo	-0.0086 (0.12)	0. 222	-0.0216 (0.10)	0.472	0.0306 (0.20)	0.910
VEGF-D	Sunitinib	0.2476 (0.26)	**<0.001**	0.1038 (0.27)	0.131	0.0914 (0.20)	0.133
	Placebo	-0.1093 (0.66)	0.616	-0.0094 (0.19)	0.811	-0.1061 (0.25)	0.281
SDF-1α	Sunitinib	0.0914 (0.47)	0.756	0.2912 (0.92)	0.730	-	-
	Placebo	-0.0827 (0.93)	0.820	0.3572 (0.85)	**0.041**	-	-

^a^Data are presented as mean (SD). *P* < 0.05 marked in boldface and considered significant using paired Wilcoxon test

**Table 4:** Association between biomarker serum levels and outcome

Serum biomarker		PFS		OS	
		Median (days)	HR (95 % CI)	Median (days)	HR (95 % CI)
			*P* value		*P* value
VEGF-A	low-level baseline	166	0.533	329	0.602
	high-level baseline	91	(0.318–0.895)	270	(0.356–1.018)
			***P*** ** = 0.017**		*P* = 0.058
sVEGFR2	low-level baseline	107	1.682	293	1.148
	high-level baseline	167	(1.014–2.789)	330	(0.648–1.927)
			***P*** ** = 0.044**		*P* = 0.601
VEGF-D	low-level baseline	123	1.670	310	0.888
	high-level baseline	159	(0.760 –3.673)	300	(0.527–1.496)
			*P* = 0.200		*P* = 0.654
SDF-1α	low-level baseline	160	0.749	300	0.783
	high-level baseline	129	(0.417–1.321)	329	(0.429–1.431)
			*P* = 0.310		*P* = 0.427

*HR* hazard ratio, *PFS* progression free survival, *OS* overall survival, *CI* confidence interval. *P* < 0.05 marked in boldface

### SDF-1α/CXCL12

Blood samples of 50 patients were available for biomarker analysis of SDF-1α. A significant increase of SDF-1α from median baseline levels of 143 pg/ml to 337 pg/ml at day 1 of cycle 2 was detected for PL only (Wilcoxon matched-pairs test *P* = 0.041). No significant benefit for PFS or OS could be shown in the small subgroup of this biomarker analysis. A trend for longer PFS in both treatment arms with an increase in SDF-1α from baseline to cycle 2 was shown (Wilcoxon matched-pairs test *P* = 0.058).

### VEGF-A

The median baseline serum level of VEGF-A was 58.7 pg/ml for 65 evaluable patients. There was a significant reduction in VEGF-A levels from baseline to day 14 of the first cycle in the SUN group (Wilcoxon matched-pairs test *P* = 0.017). Interestingly, we detected a significant decrease of VEGF-A levels from baseline to day 1 of cycle 2 for the PL group only (*P* = 0.033). For the PL group, a similar trend towards reduction of VEGF-A levels from baseline to day 1 of cycle 3 could be shown (*P* = 0.062). In contrast, no change between baseline and day 1 of cycle 2 could be shown for patients receiving SUN. Moreover, this result was supported by a trend to an increase in VEGF-A concentration from day 14 of cycle 1 to predose measurement on day 1 of cycle 2 for the SUN group only (*P* = 0.10). A VEGF-A reduction from baseline to day 14 of cycle 1 correlated positively with longer OS in the PL group only (HR 0.286, 95 % CI 0.101–0.814, *P* = 0.019).

**Fig. 3:**
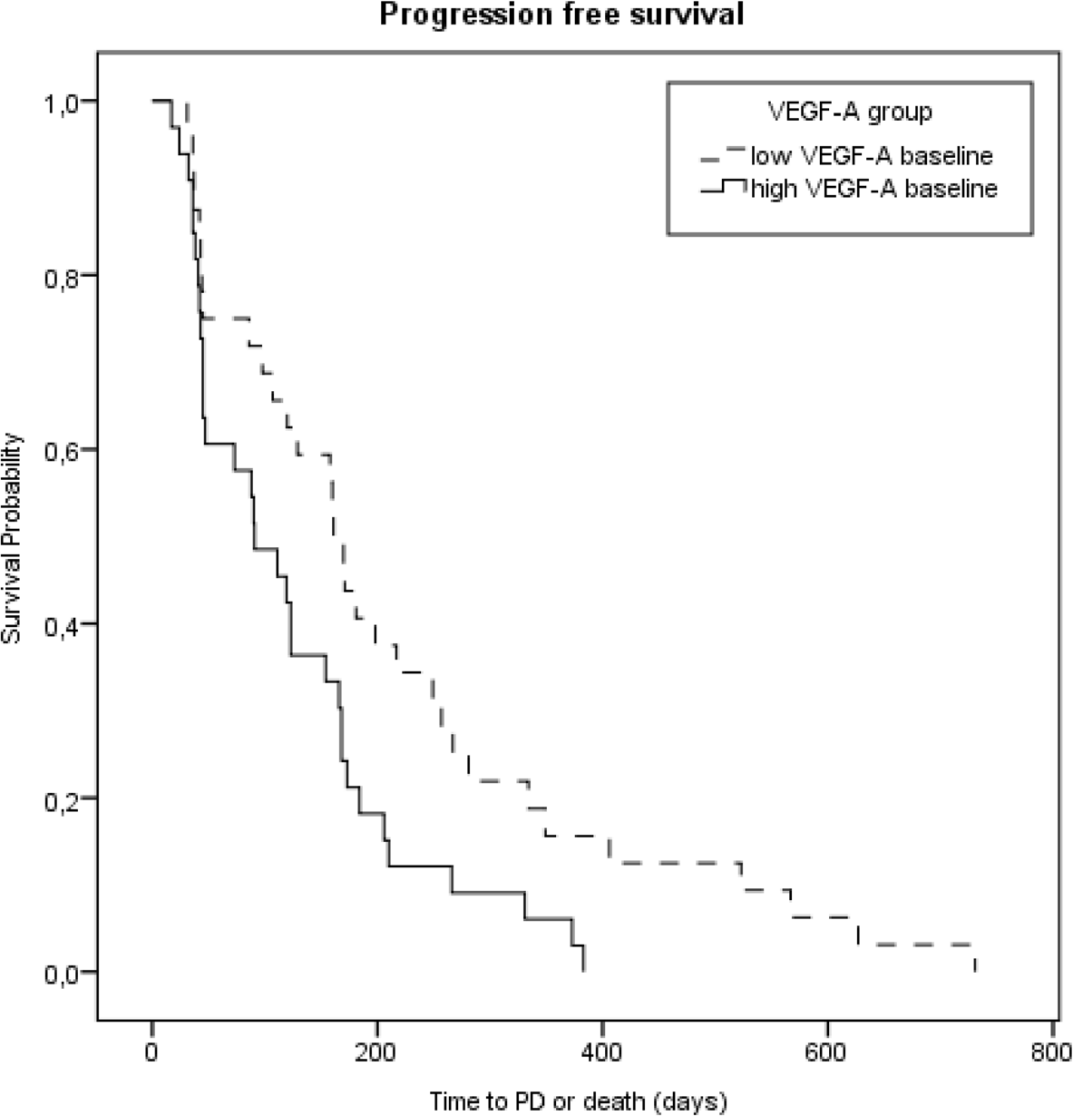
Kaplan-Meier curves for progression-free survival depending on VEGF-A at baseline dichotomized at the median 58.7 pg/l. Hazard ratio 0.533, 95 % CI 0.318–0.895, *P* = 0.017

Independent of treatment, low VEGF-A baseline values were significantly associated with longer PFS (Fig. 3). Additionally, a strong trend to a longer OS of 11.07 months ± 63 days for the group with low VEGF-A baseline levels versus 7.56 months ± 73 days (HR 0.602, 95 % CI 0.356–1.018, *P* = 0.058) was detected.

### sVEGFR2

**Fig. 4:**
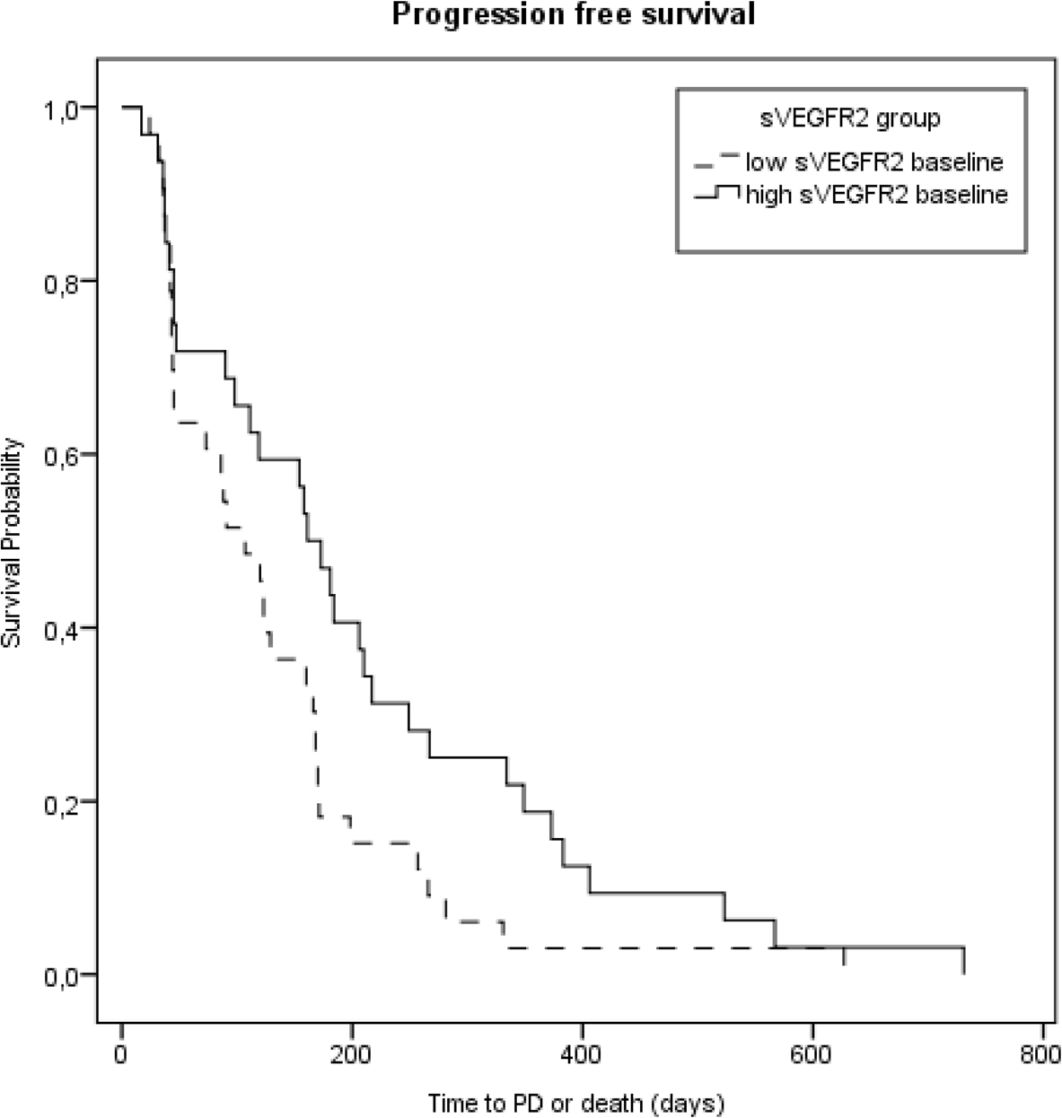
Kaplan-Meier curves for progression-free survival depending on sVEGFR2 at baseline dichotomized at the median 4688 pg/ml. Hazard ratio 2.557, 95 % CI 1.248–5.237, *P* = 0.010

The median concentration of sVEGFR2 prior to the start of study treatment was 4688 pg/ml for 65 evaluable patients. Analysis of serum values showed significantly decreased sVEGFR2 after 14 days drug intake during cycle 1 in the SUN group (Wilcoxon matched-pairs test *P* = 0.012). A change from a median level of 4613 pg/ml to 4197 pg/ml after 14 days was observed. Furthermore, concentrations on day 1 of cycle 2 were significantly lower than baseline in the SUN group (4238 pg/ml, Wilcoxon matched-pairs test *P* = 0.006). Between baseline and day 1 of cycle 3 a similar trend for a total of 18 available patient samples with a median concentration of 4312 pg/ml could be shown (*P* = 0.078). In contrast no significant level changes could be determined for patients in the PL group. High sVEGFR2 baseline levels significantly correlated with a longer PFS (HR 1.682, 95 % CI 1.014–2.789, *P* = 0.044). Additionally, we found a relationship between high sVEGFR2 levels on day 1 of cycle 2 and longer PFS independent of treatment (HR 2.557, 95 % CI 1.248–5.237, *P* = 0.010) as shown in Fig. 4. However for OS, no association of sVEGFR2 was found in the analyses.

### VEGF-D

Serum samples from 64 patients with a median baseline concentration of 712.4 pg/ml were available for analysis of VEGF-D. The comparison of VEGF-D levels between baseline and day 14 of cycle 1 showed a significant increase in the SUN group (Wilcoxon matched-pairs test with median values of 681 pg/ml to 913 pg/ml, *P* < 0.001), but no change in the PL group. Furthermore, a similar trend to higher VEGF-D levels on day 1 of cycle 2 was shown for patients receiving SUN (681 pg/ml to 807 pg/ml, *P* = 0.19).

**Fig. 5:**
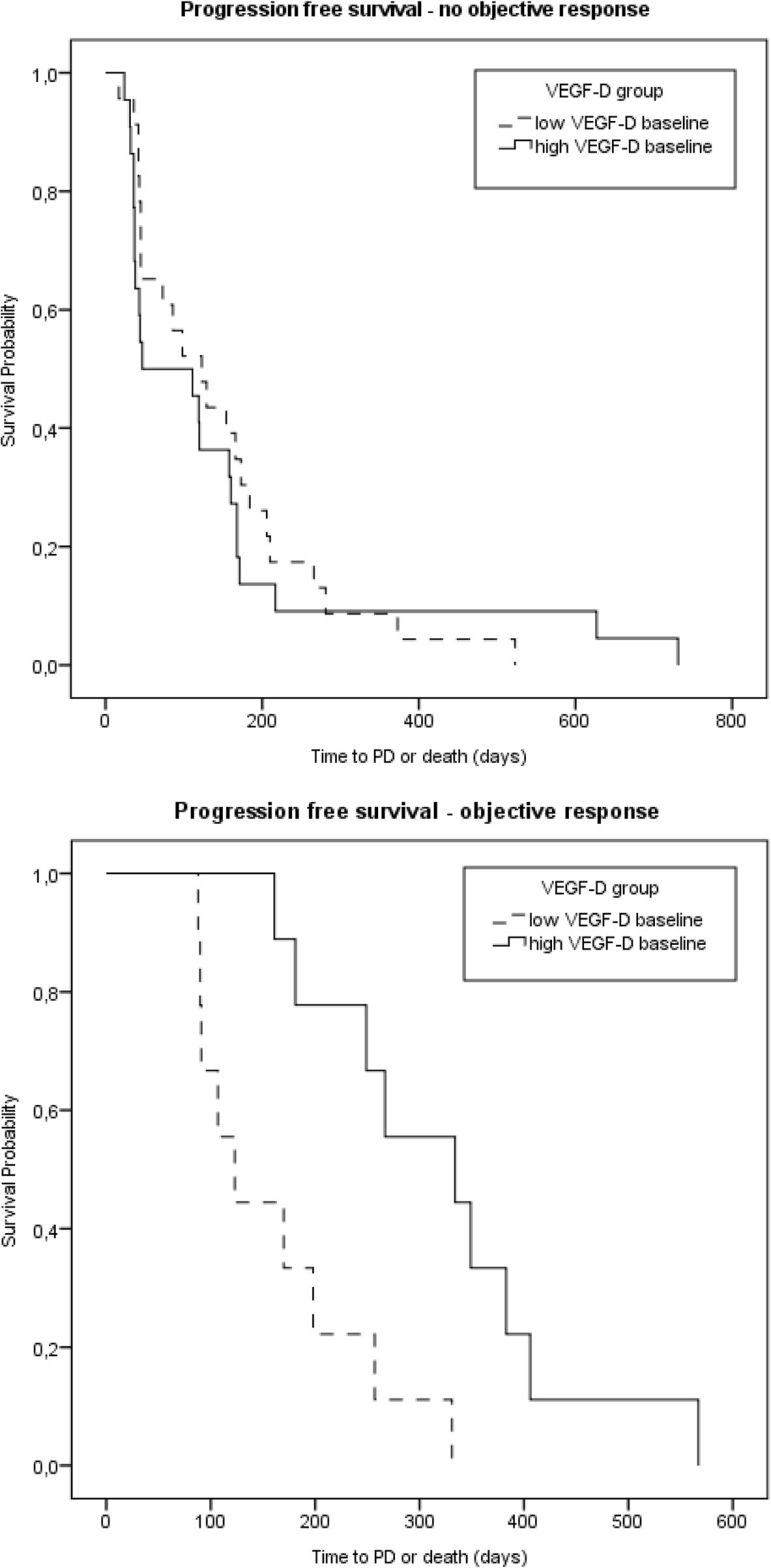
Kaplan-Meier curves for progression-free survival depending on VEGF-D baseline and objective response dichotomized at the median >712 pg/l. Patients without objective response: Hazard ratio for VEGF-D: 1.192, 95 % CI 0.657–2.164, *P* = 0.56, *N* = 46. Patients with objective response: Hazard ratio for VEGF-D: 0.189, 95 % CI 0.056–0.637, *P* = 0.007, *N* = 18

The population was then stratified into two groups depending on best response: 46 patients without objective response (stable disease or progression) and 18 patients with a proven objective response (complete or partial response). For patients with an objective response, high VEGF-D baseline concentrations were associated with a longer PFS independent of treatment (11.1 vs. 4.1 months, HR 0.189, 95 % CI 0.056–0.637, *P* = 0.007; Fig. 5). An analysis following patient separation into quartile groups according to their VEGF-D baseline concentration confirmed that the longest median PFS for patients were associated with the highest baseline values above the 75 percentile (HR 1.801, 95 % CI 1.033–3.141, *P* = 0.038). Moreover, high VEGF-D levels on day 1 of cycle 2 also correlated with significantly longer PFS in the group with objective response (HR 4.236, 95 % CI 1.226–14.638, *P* = 0.023). Similar to sVEGFR2 however, no association of VEGF-D with OS was found, in the analyses.

## Discussion

Patients with chemorefractory AGC have a poor prognosis. After failure of first-line treatment, various options for second-line treatment have been analyzed in previous studies, but median OS has always remained below 10 months. Recent randomized phase III trials of second-line chemotherapy versus BSC have reported even lower OS times [6, 7, 31]. Here, FOLFIRI has been considered to be an established option after failure of a platinum-containing first-line therapy [2]. Other TKIs such as erlotinib and gefitinib have been under investigation in the first- and second-line treatment of gastric cancer [17, 32, 33]. Ramucirumab, a fully humanized monoclonal antibody targeting VEGFR2, recently showed a significant OS benefit as second-line monotherapy and in combination with paclitaxel [34, 35].

In our study, a beneficial effect of SUN added to FOLFIRI on the endpoints PFS, OS and duration of disease stabilization could not be verified. The primary endpoint, PFS, was similar for FOLFIRI + SUN versus FOLFIRI + PL. Regarding OS however, patients receiving SUN had a trend towards a better OS with a median OS of 315 versus 270 days compared with PL. In terms of OS, the benefit of second-line therapy versus BSC has already been demonstrated in trials such as those of Thuss-Patience et al. and Kang et al. [6, 7]. Two previous second-line, phase II studies with SUN alone reported medians for PFS of 1.3 and 2.3 months and for OS of 5.8 and 6.8 months, respectively [28, 29]. Even if cross study comparison has limitations, FOLFIRI combined with SUN showed an additional improvement with respect to those endpoints compared to SUN or FOLFIRI alone.

Generally, the combination of SUN and FOLFIRI was well tolerated. No unexpected toxicities occurred in patients receiving SUN, demonstrating the safety of such a second-line combination. These data are consistent with a phase I dose-escalation study of the FOLFIRI + SUN regimen [36] and a study of SUN alone in patients with chemorefractory AGC. With respect to non-hematological AEs, diarrhea, vomiting, and lethargy were also most frequent in patients being treated with the higher dose of 37.5 mg/day SUN [36]. The higher frequency of non-hematological toxicities observed in the current study may be attributed to the backbone chemotherapy or underlying disease, characterized by the weaker general condition of second-line AGC patients [28]. Similar studies investigating FOLFIRI in patients with AGC have reported neutropenia, anemia, nausea, diarrhea, and stomatitis as common toxicities as observed in our study [37, 38]. The FOLFIRI combination with daily 37.5 mg SUN versus PL has also been investigated in patients with metastatic colorectal cancer (mCRC) [39]. This phase III study was stopped prematurely due to the occurrence of more grade ≥3 AEs with SUN than PL and a high incidence of toxicity-related deaths. However, these differences between the treatment arms were less marked in our study compared with those observed in patients with mCRC. Since toxicity-related deaths were also less frequent, the lower daily dose of 25 mg SUN may be a more attractive dose level for combination with FOLFIRI. As indicated by Park et al. [40] quality of life (QoL) should be a consideration when considering second-line therapies. Based on the EORTC QLQ-C30 and HADS questionnaires, QoL in our trial was nearly always in favor of SUN (data not shown).

Serum was used for biomarker analysis in our study. No adjustment of the platelet factor was performed for the biomarker concentrations. Both serum and plasma VEGF levels have been reported as prognostic biomarker for survival in gastric cancer and other tumors [12, 41]. Nevertheless, the appropriate specimen to analyze have been discussed extensively in previous literature. For instance Lee et al. suggested serum analysis for VEGF level determination even being affected by platelet-derived VEGF [42, 43].

With regard to the tested biomarkers in our analysis, SUN induced a significant decrease in sVEGFR2 concentration during the first 6 weeks of treatment. Interestingly, higher sVEGFR2 levels during the first 6 weeks of study treatment indicated a longer PFS independent of treatment group. With respect to VEGF-A serum levels, firstly a decrease was observed after 14 days treatment combined with a VEGF-A increase after treatment rest in the SUN group. Additionally, an increase in VEGF-D levels in SUN-treated patients during the first 6 weeks of therapy was observed. High VEGF-A baseline levels were prognostic for shorter PFS in both study groups. Additionally, a trend towards shorter OS was shown for patients with a high VEGF-A baseline. A significant improvement in PFS or OS with respect to VEGF-D levels was noted for patients with objective response during treatment only. These findings confirm previous investigations suggesting VEGF-A, −D and sVEGFR2 as potential prognostic biomarkers for therapy-resistant patient outcome [12, 44]. Here, our results highlight again the complexity of the pathomechanisms and the difficulties in the treatment of GC, as no identified biomarker level change during SUN treatment directly correlated with response or survival benefit [45, 46].

## Conclusion

In summary, although FOLFIRI + SUN demonstrated positive trends in overall survival times, the study did not meet its primary endpoint. The median PFS was similar in both groups. Nevertheless, to our knowledge, no biomarker results under these treatment regimens have been published to show the effects on in-vivo angiogenesis. Thus, novel therapy concepts targeting sVEGFR2 and other VEGF group members to normalize vascularization in therapy-resistant tumor tissue should be developed together with biomarkers, such as serum or protein expression levels during anticancer treatment [47].

## Additional files

Additional file 1: Inclusion and exclusion criteria of the SUN-CASE clinical trial protocol. (DOCX 16 kb) Additional file 2: Ethics Committees that approved the SUN-CASE clinical trial. (DOCX 18 kb)

## Abbreviations

AIO: Arbeitsgemeinschaft Internistische Onkologie
