# Supplementary material for: Sunitinib added to FOLFIRI versus FOLFIRI in patients with chemorefractory advanced adenocarcinoma of the stomach or lower esophagus: a randomized, placebo-controlled phase II AIO trial with serum biomarker program
Source: BMC Cancer. 2016 Aug 31;16(1):699. doi: 10.1186/s12885-016-2736-9 (PMC5006426; doi:10.1186/s12885-016-2736-9)
Supplement: Additional file 1: — Inclusion and exclusion criteria of the SUN-CASE clinical trial protocol. (DOCX 16 kb) [file 12885_2016_2736_MOESM1_ESM.docx]

**Additional file 1: Table S1: Inclusion and exclusion criteria of the SUN-CASE clinical trial protocol**

**Inclusion Criteria:**

• Signed and dated informed consent before the start of specific protocol procedures

• Histological proven gastric adenocarcinoma including adenocarcinoma of the esophagogastric junction or lower esophagus

• Failure of any prior palliative chemotherapy (docetaxel and/or platinum-based chemotherapy); but patient has not previously received FOLFIRI treatment

• Measurable metastatic disease according to the RECIST criteria. If locally recurrent disease, it must be associated with at least one measurable lymph node (> 20 mm by CT scan or > 10 mm with spiral CT)

• Age: ≥ 18 years

• Karnofsky index 100 – 70 %

• Life expectancy > 12 weeks

• Adequate hematological, hepatic and renal functions

• At least 3 weeks from previous docetaxel– and/or platinum-based chemotherapy

• Recovery from side effects of any prior therapy

• Able to comply with scheduled assessments and with management of toxicity

**Exclusion criteria:**

• History of another primary malignancy ≤3 years, with the exception of nonmelanoma skin cancer and in situ carcinoma of the uterine cervix

• Any prior palliative radiotherapy

• Concurrent treatment with any other anti-cancer therapy

• Prior treatment with a VEGF, VEGFR or RTK inhibitor, or prior enrolment on this study

• Known allergic/hypersensitivity reaction to any of the components of the treatment

• Treatment with potent CYP3A4 inhibitor within 7 days of Sunitinib/placebo dosing or with potent CYP3A4 inducer within 12 days of Sunitinib/placebo dosing

• Other serious illness or medical conditions within the last 12 months prior to study drug administration

• Unstable cardiac disease despite treatment; myocardial infarction within 12 months prior to study entry; congestive heart failure NYHA grade 3 and 4

• Hypertension that cannot be controlled by medication (>150/100 mmHg) despite optimal medical therapy

•Ongoing cardiac dysrhythmias of NCI CTCAE grade ≥2, atrial fibrillation of any grade, or QTc interval >450 msec for males or >470 msec for females

• History of significant neurologic or psychiatric disorders including dementia or seizures

• Active uncontrolled infection

• History of clinically significant bleeding within the past 6 months, including hemoptysis or hematuria, or underlying coagulopathy

•Active disseminated intravascular coagulation

•Deep vein thrombosis, or other significant thromboembolic event

• Cerebrovascular accident including transient ischemic attack

•Pulmonary embolus

• Bowel obstruction or chronic diarrhea, history or presence of inflammatory enteropathy or extensive intestinal resection (> hemicolectomy or extensive small intestine resection with chronic diarrhea), Crohn's disease, ulcerative colitis

•Peptic ulcer disease

• History of abdominal fistula, gastrointestinal perforation, or intra-abdominal abscess within 6 months prior to study enrolment, unless affected area has been removed surgically

• Known deficit in DPD

• Hypercalcemia not controlled by bisphosphonates

• Contraindications to the use of atropine

• Current treatment with therapeutic doses of anticoagulant medication (low dose warfarin or equivalent up to 2 mg PO daily for deep vein thrombosis prophylaxis is allowed)

• Pregnant or lactating women; female patients who are pregnant or lactating or men and women of reproductive potential not willing or not able to employ an effective method of birth control/contraception to prevent pregnancy during treatment and for 3 months after discontinuing study treatment

• Known drug abuse/alcohol abuse

• Current, recent, or planned participation in an experimental treatment drug study other than this protocol

• Major surgical procedure, open biopsy or significant traumatic injury within 4 weeks before starting treatment; anticipation of need for major surgical procedure (e.g. impending bowel obstruction) during the course of the study

• History of other medical or psychiatric condition, metabolic dysfunction, physical examination finding, or clinical laboratory finding giving reasonable suspicion of a disease or condition that contraindicates the use of an investigational drug or that might affect the interpretation of the results of the study or render the patient at high risk from treatment complications
