# Supplementary material for: Sunitinib added to FOLFIRI versus FOLFIRI in patients with chemorefractory advanced adenocarcinoma of the stomach or lower esophagus: a randomized, placebo-controlled phase II AIO trial with serum biomarker program
Source: BMC Cancer. 2016 Aug 31;16(1):699. doi: 10.1186/s12885-016-2736-9 (PMC5006426; doi:10.1186/s12885-016-2736-9)
Supplement: Additional file 2: — Ethics Committees that approved the SUN-CASE clinical trial. (DOCX 18 kb) [file 12885_2016_2736_MOESM2_ESM.docx]

**Additional file 2: Ethics Committees that approved the SUN-CASE clinical trial**

| **Ethics Committee** |
| --- |
| Ethics Committee of the Medical Association of Rhineland-Palatinate, Mainz |
| Ethics Committee of the Medical Association of Hamburg |
| Ethics Committee of the Medical Association North Rhine, Düsseldorf |
| Ethics Committee of the Medical Faculty of the University of Rostock |
| Ethics Committee of the Bavarian Medical Association, Munich |
| Ethics Committee of the Medical Faculty of the University of Würzburg |
| Ethics Committee of the Medical Association of the Saarland, Homburg |
| Ethics Committee of the Medical Faculty of the Technical University of Munich |
| Ethics Committee of the Medical Faculty of the Martin-Luther University Halle-Wittenberg |
| Ethics Committee II of the Medical Association of Schleswig-Holstein, Bad Segeburg |
| Ethics Committee of the University of Ulm |
| Ethics-Committee at the State Office for Health and Social Affairs Berlin |
| Ethics Committee of the Medical Faculty of the University of Regensburg |
| Ethics Committee of the Medical Association of Westphalia-Lippe and the Medical Faculty of the University of Münster |
| Ethics Committee of the Medical Association of Hesse, Frankfurt am Main |
| Ethics Committee of the Medical Association of Baden-Württemberg, Stuttgart |
| Ethics Committee of the Medical Faculty of the University of Duisburg-Essen |
